# Molecular Characterization and Analysis of Human *Trichostrongylus* Species in an Endemic Region of Iran Based on *COX* 1 Gene; A Cross‐Sectional Study

**DOI:** 10.1002/hsr2.70612

**Published:** 2025-04-18

**Authors:** Sara Nemati, Hanieh Mohammad Rahimi, Meysam Sharifdini, Hamed Mirjalali

**Affiliations:** ^1^ Foodborne and Waterborne Diseases Research Center, Research Institute for Gastroenterology and Liver Diseases Shahid Beheshti University of Medical Sciences Tehran Iran; ^2^ Department of Medical Parasitology and Mycology, School of Medicine Guilan University of Medical Sciences Rasht Iran

**Keywords:** cytochrome oxidase gene, Iran, phylogenetic analysis, *Trichostrongylus*, zoonotic infection

## Abstract

**Background and Aims:**

*Trichostrongylus* species are the causative agents of zoonotic disease, which has been frequently reported from animals in Iran. The aim of this study was to identify, molecular characterization and analysis of *Trichostrongylus* species isolated from humans, in an endemic region, based on cytochrome c oxidase (*COX) 1*.

**Methods:**

A total of 206 fresh stool samples were collected from residents of endemic villages of sampling area. All samples had been examined using conventional parasitological methods, along with the PCR technique. After amplification and sequencing of a discriminative region of *COX1* gene, the phylogeny relationship, haplotype network, and molecular diversity between *Trichostrongylus* spp., were scrutinized using PopART networking, DnaSP v.6, and MEGA10 software.

**Results:**

In total, from 206 fecal samples, 71 people (34.4%) were infected with *Trichostrongylus* spp. The ~700‐bp fragment of the *COX*1 was amplified in all 71 morphological positive samples, however, 33 samples were successfully sequenced, belonging to *Trichostrongylus* spp. In this study, *T. colubriformis* was the predominant species and one sequence was characterized as *T. vitrinus*. Our sequences were grouped together with sequences, which were obtained from animals in the same region (97.17% similarity). In total, 26 haplotypes were identified and haplotype diversity ranged from 0.988 ± 0.012.

**Conclusions:**

In view of the importance of Trichostrongylosis to public health as zoonotic infection, information about its prevalence in animal and human populations can provide valuable information on how different types of this parasite are transmitted between people and animal's host.

## 1 Introduction

Among ruminants, *Trichostrongylus* spp., are common gastrointestinal parasitic pathogens with a global spread [1, 2, 3, 4, 5, 6]. These nematodes provide serious health risks, reduce animal products and in severe forms may lead to death in animals [7, 8, 9]. Trichostrongilosis is generally asymptomatic and little is known about its pathogenesis and zoonotic aspects. The clinical manifestations of infection in humans range from mild (malaise, generalized fatigue, dizziness, flatulence, diarrhea, nausea, and abdominal pain) to severe symptoms (anemia, cholecystitis, and emaciation). In severe forms of infection, *Trichostrongylus* can cause mild anemia [10, 11, 12]. The clinical symptoms in human are generally not severe; however, some people may complain from gastrointestinal discomfort and eosinophilia [3].

Due to the importance of zoonotic diseases, and the close relationship between human with animal [3, 13], *Trichostrongylus* spp. represented a major public health problem over the world [14]. Application of nonchemical fertalizers such as night‐soils increases the risk of transmission of this nematod to humans [3]. Human as incidental hosts, become infected upon ingestion of infective filariform (L3) larvae of the parasite in contaminated food or water resources [3, 6, 15]. Until now, 12 species for *Trichostrongylus* spp. have been reported from humans [1, 12, 16]. The human infections in the Middle East are mostly assigned to *T. colubriformis* and *T. orientalis* [17, 18]. The prevalence of *Trichostrongylus* infection in the general population in Iran is 10% (95% CI: 1.6% to 17%) [19], and Fuman city is considered as an endemic region for human trichostrongylosis [18].

For the phylogenetic investigation of *Trichostrongylus* and the genetic variation within it, molecular analyses based on the internal transcribed spacer (ITS) and 28S region of the ribosomal DNA (rDNA) genes were employed [2, 5, 16, 20]. Although most of studies have employed ITS2 fragment to analyze the genetic variations and phylogenetic relationships of the *Trichostrongylus* spp. [14, 21], mitochondrial (mt) fragments can provide more data [22, 23, 24]. The mt genes (mitogenome) are highly conserved genetic fragments, which makes them a valuable resource to study the DNA structure and the evolutionary and phylogenetic relationships of species [25]. Accordingly, few studies have employed mtDNA to identify species of the *Trichostrongylidae* family [26, 27], and there are few publications on the mt gene of nematodes from Iran [14, 28, 29]. In this regard, the current research aimed to characterize and phylogenetically analyze the cytochrome c oxidase subunit I (*COX*1) from the mitochondrial gene of *Trichostrongylus* species in human samples collected from an endemic region in north of Iran.

## 2 Methods

### 2.1 Ethical Approval

This project was approved by the Ethical Review Committee of the Research Institute for Gastroenterology and Liver Diseases, Shahid Beheshti University of Medical Sciences, Tehran, (IR. SBMU. RIGLD. REC.1400.011).

### 2.2 Samples and Study Area

**Figure 1:**
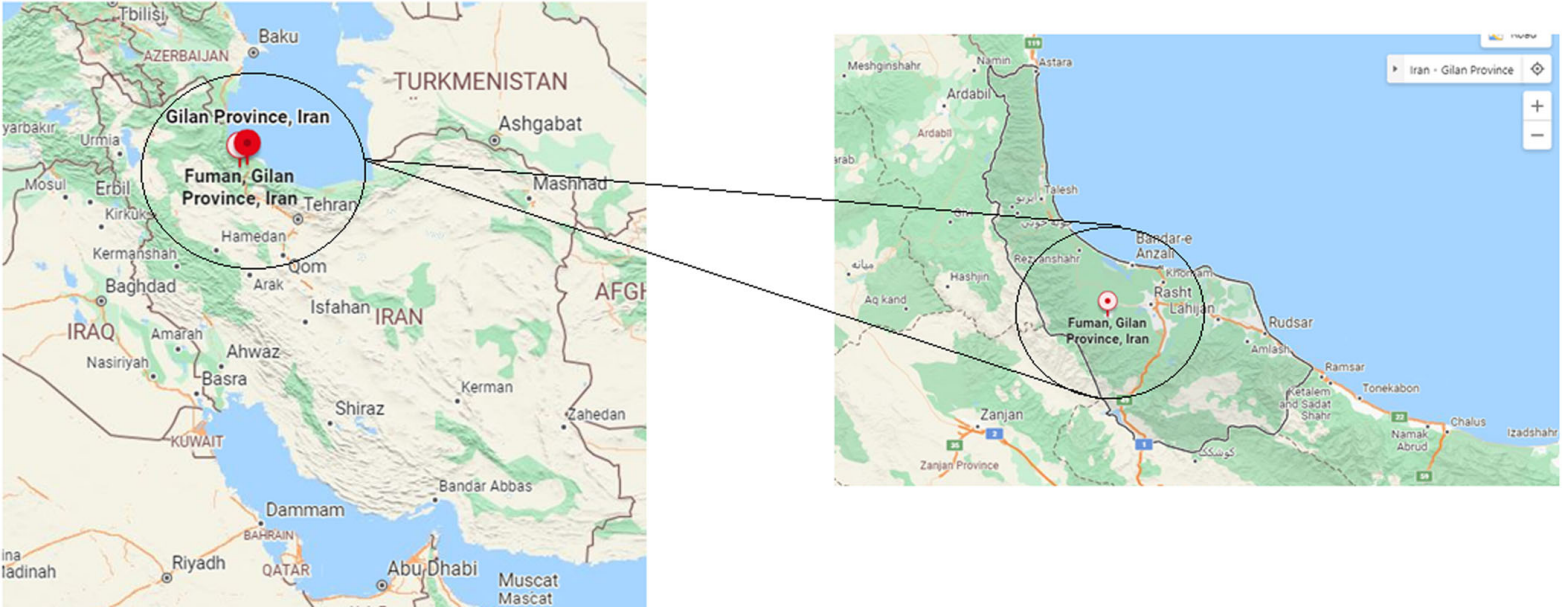
Geographical map of Guilan province, Fuman district, North of Iran.

A total of 206 fresh stool samples were collected from villagers in Guilan province, northern Iran, from June to October 2020. The stool samples collected were immediately transferred to the Department of Parasitology and Mycology, Guilan University of Medical Sciences. Guilan has a humid subtropical climate with, by a large margin, the heaviest rainfall in Iran: reaching as high as 1900 millimeters (75 in) in the southwestern coast and generally around 1400 millimeters (55 in), which all provide a suitable condition for transmission of soil transmitted helminths (STHs). Fuman County is in Gilan province, in northwestern Iran; its capital is the city of Fuman. It is only 21 km to the west‐southwest of Rasht, the capital of the province, and 356 km away from the national capital Tehran (Figure 1).

### 2.3 Genomic Characterization

In this study, identification of *Trichostrongylus* spp. was determined based on the morphological characters and flotation methods [30]. Accordingly, 2–3 g of stool samples were floated in 20 mL of saturated sodium chloride solution in a test tube with a coverslip on the top. After 15 min, the coverslip was lifted and screened by a light microscopy (Willis technique). All samples, which were parasitologically positive for *Trichostrongylus* spp., were directed to molecular investigation. For this purpose, a commercial DNA extraction kit (Yektatajhiz, Tehran, Iran) was employed to extract total DNA from stool samples, which were identified positive for *Trichostrongylus* spp. Briefly, the supernatant of Willis method was collected, washed twice with distilled water, and introduced to the DNA extraction process. Extracted DNA was stored at −20°C for PCR amplification.

The identical fragment of the *COX*1 gene, with approximately 700 bp length, was amplified using the LCO1490 (5′‐GGTCAACAAATCATAAAGATATTGG‐3′) and HCO2198 (5′‐TAAACTTCAGGGTGACCAAAAAATCA‐3′) primers. The reaction mixture, conditions, and protocol for the polymerase chain reaction amplification were done based on the previously mentioned study [31]. The PCR amplification was performed in 20 µl volumes containing 2X red PCR premix (Ampliqon, Odense, Denmark), 10 pmol of each primer, and 3 µl of extracted DNA (20–50 ng). The thermal PCR protocols included an initial denaturation step at 95°C for 5 min followed by 35 cycles of denaturation at 95°C for 40 s, annealing at 54°C for 90 s, an initial extension step at 72°C for 60 s, and a final extension step at 72°C for 10 min. PCR products were visualized by electrophoresis using 1.5% agarose, and staining with 7 μl/100 ml of ethidium bromide. All PCR products resulted from the amplification of identical fragment in stool samples were directly sequenced using by a 3130 ABI sequencer.

### 2.4 Sequencing, Phylogenetic and Networking Analyzing

Sequencing of the PCR products was performed by Sanger sequencing method, which was carried out in an automated DNA analyzer (ABI 3730XL, Applied Biosystems, USA). All generated sequences were manually trimmed and edited using BioEdit and using Chromas (version 2.6) software, and compared to the GenBank database through basic local alignment search tool (https://blast.ncbi.nlm.nih.gov/Blast.cgi) to characterize genus and species. All sequences were deposited to the GenBank database with accession numbers: OR429439‐OR429471.

To illustrate the phylogeny relationship between *Trichostrongylus* spp., the Molecular Evolutionary Genetics Analysis (MEGA) X software was engaged. The software suggested the best phylogenetic tree model based on the Bayesian information criterion (BIC). In this regard, phylogenetic tree for our sequences together with reference sequences, retrieved from the GenBank database, was constructed by the Maximum Likelihood (ML) and Tamura 3‐parameter incorporated in the MEGAX software. The *COX*1 sequence topologies were evaluated using 1000 bootstrap replicates to assess the significance level of the tree. The relationships in the haplotype network and molecular diversity were inferred through Templeton, Crandall and Sing (TCS) algorithm [32] using the PopART (Population Analysis) networking and DnaSP v.6 software, respectively [33].

All reference sequences, based on the *COX*1 gene, were retrieved from the GenBank database, which were associated to *T. colubriformis* (*n* = 14) and *T. axei* (*n* = 9) of ruminants from Brazil, and *T. vitrinus* (*n* = 2), *T. colubriformis* (*n* = 29) and, *T. axei* (*n* = 3) of ruminants from same location with ours in Guilan province, Iran.

## 3 Results

### 3.1 Ethics Approval and Consent to Participate

This project was approved by the Ethical Review Committee of the Research Institute for Gastroenterology and Liver Diseases, Shahid Beheshti University of Medical Sciences, Tehran, (IR. SBMU. RIGLD. REC.1400.011). A verbal informed consent was obtained from participants or their legal guardian (s), at the time of sampling.

### 3.2 Sample Identification

**Figure 2:**
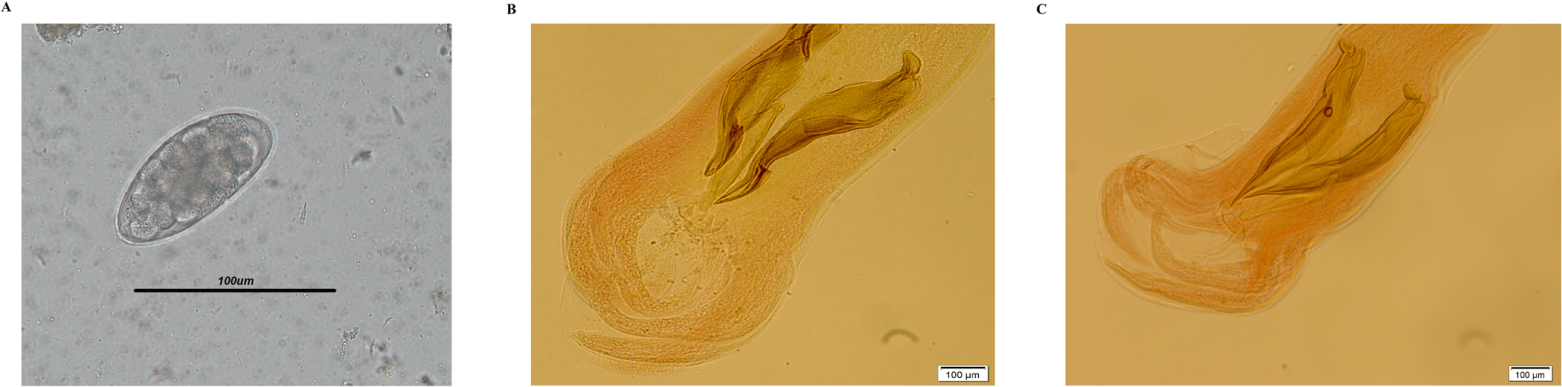
A microscopy figure of (A) the egg of *Trichostrongylus* spp. and the identical spicule of (B) *T. colubriformis* and (C) *T. vitrinus*.

In total, from 206 fecal samples, 71 samples were identified positive for *Trichostrongylus* spp. Pandi et al., [30]. The Willis floatation method coupled with microscopy showed the presence of identical eggs for *Trichostrongylus* spp., sized 75–110 µm in samples. The filariform larvae of the parasite was seen in agar plate cultivation of infected stools [30] (Figure 2). A ~700‐bp fragment of the *COX*1 was amplified in all 71 morphological positive samples, however, 33 samples were successfully sequenced and belonged to the genus of *Trichostrongylus*. An identity more than 95% to the available sequences, previously submitted to the GenBank database, was considered as a good sequence. Unfortunately, the other 38 samples were sequenced that were failed. The reason for this result was unspecific or faint amplification of the fragment. In this study area, *T. colubriformis* was the predominant species. Accordingly, 32 and one sequences were characterized as *T. colubriformis* and *T. vitrinus*, respectively.

### 3.3 Phylogenetic Analysis

**Figure 3:**
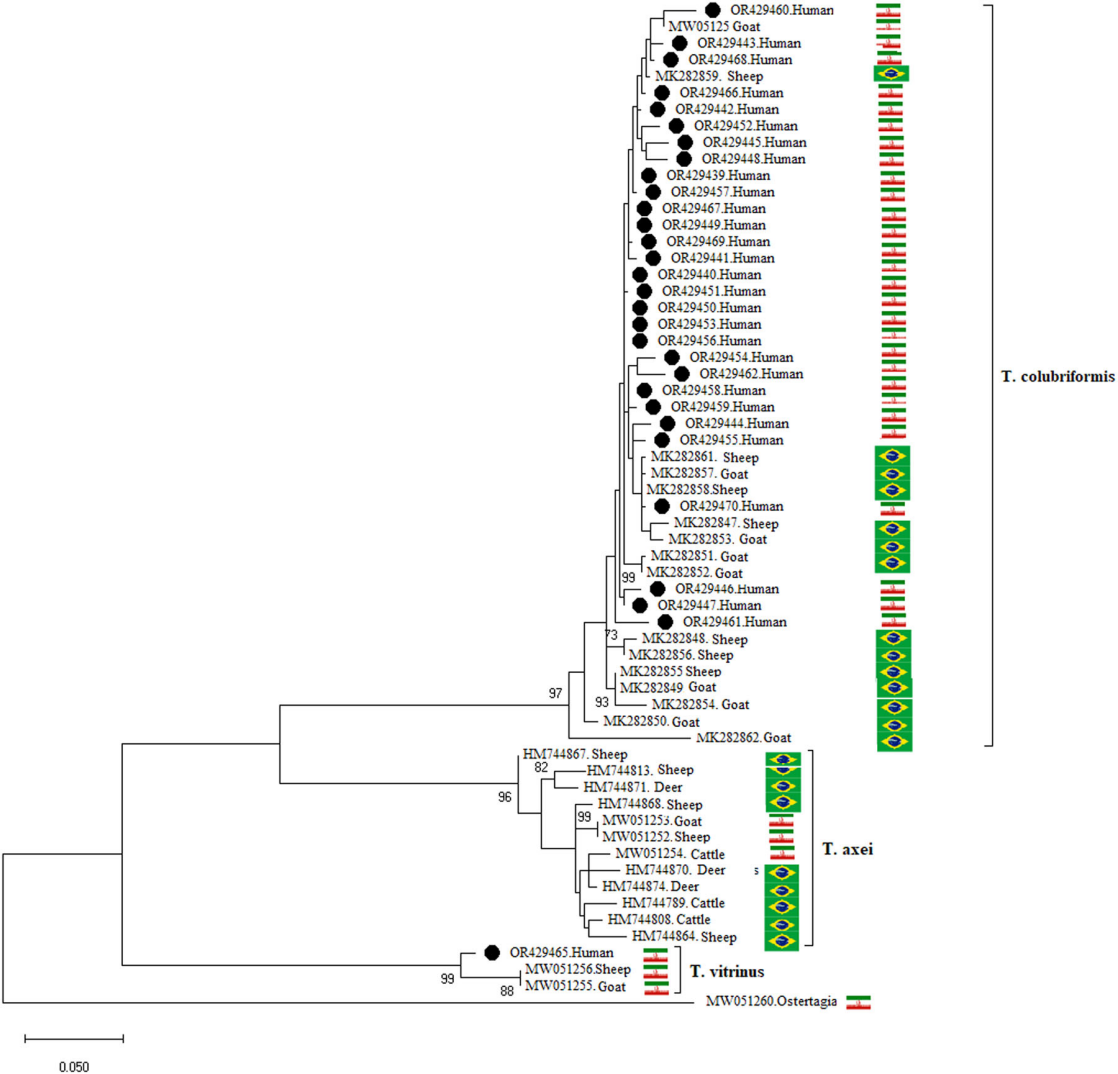
The phylogenetic tree of *Trichostrongylus* spp., was constructed based on the maximum‐likelihood and Tumura 3‐parameter algorithms. The *COX*1 sequence topologies was evaluated using 1000 bootstrap replicates. This phylogenetic tree shows similarity between the *Trichostrongylus* spp. sequences in our study with those isolated from animals.

The ML phylogenetic tree showed that the *Trichostrongylus* spp. sequences were categorized into three main groups including *T. colubriformis*, *T. vitrinus*, *T. axei*. The clade *T. colubriformis* consisted of our 29 sequences of *T. colubriformis* together with reference sequence, which was related to isolates from goat with acc no. MW051250 from Giulan province, Iran (identity: 99.06%). In addition, all reference sequences isolated from goats and sheep from Brazil were clustered with our sequences. Our *T. vitrinus* sequence was grouped together with *T. vitrinus* sequences, which were obtained from goat and sheep in Guilan province, Iran (97.17%) similarity with reference sequence: MW051255, MW051256 (Figure 3).

### 3.4 Nucleotide Diversity and Networking Analysis

In this study, nucleotide diversity of our *T*. *colubriformis* sequences (*n* = 29) was analyzed. The estimation of the numbers of synonymous (*d*_S_) and nonsynonymous (*d*_N_) substitutions per site between two sequences showed, *d*_S_: 122.05; Pi(s): 0.21509; and *d*_N_: 405.95; Pi(a): 0.01835. Moreover, for measurement of codon usage bias values, effective number of codons (ENC) parameter 36.259 were estimated. A not statistically significant trend toward a negative Tajima's D was observed in sequences (Tajima's D: –1.54772; Not significant, *p* > 0.10) (Table 1).

**Table 1:** Genetic diversity parameters of *T. colubriformis*.

Number of sequences used	Number of sites	Invariable sites	Segregating	H*	Hd*	Pi*	Tajima's D	S*	N*
29	530	484	46	25	0.983	0.01323	–1.54772	122.99	405.01

Abbreviations: H, haplotypes; Hd, haplotype diversity; S, synonymous sites/N, nonsynonymous sites; Pi, nucleotide diversity.

**Figure 4:**
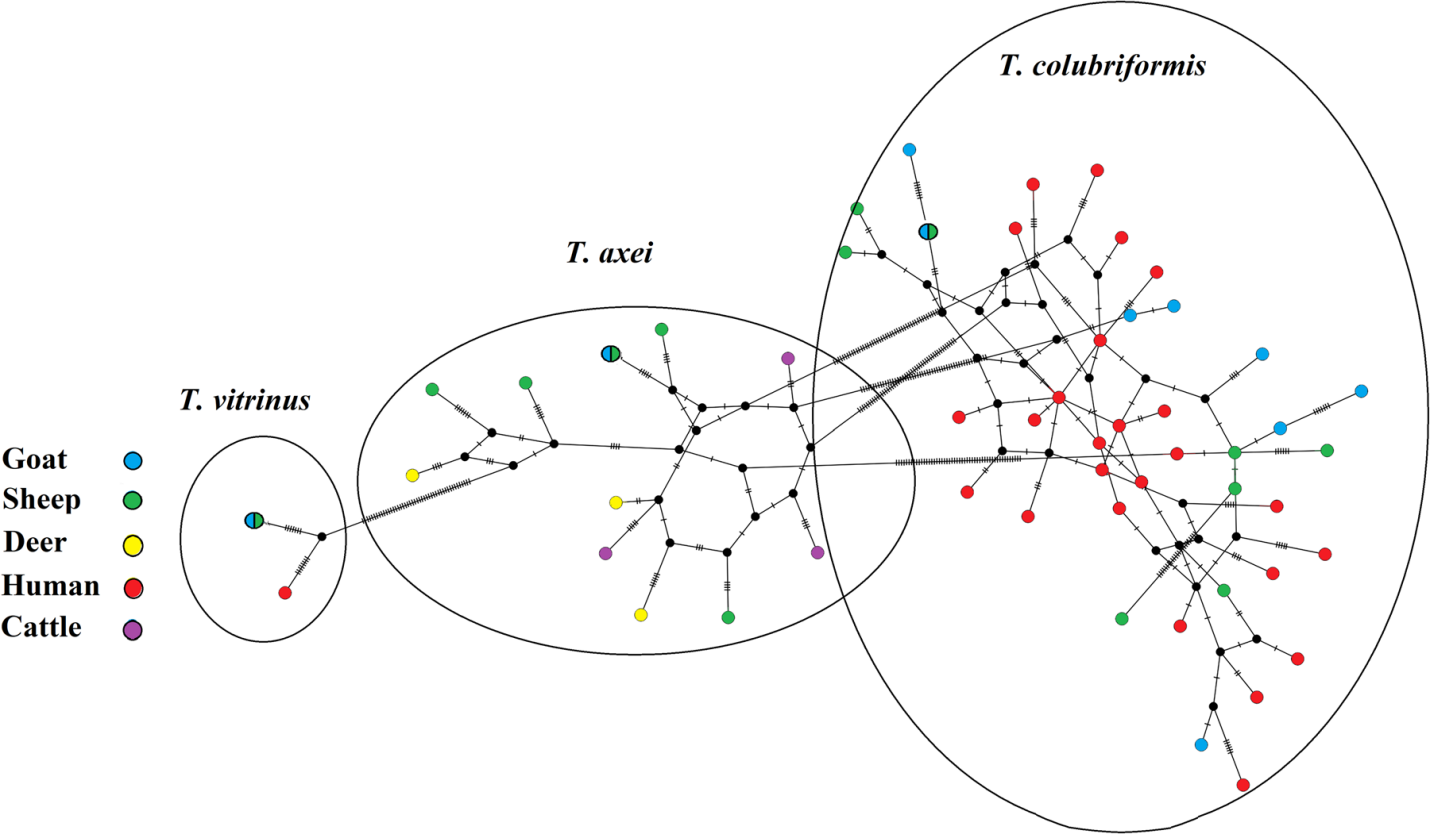
TCS sequence‐type network generated for the *COX*1 gene. The size of the circle indicates the relative frequency of the sequences belonging to a particular sequence type. Hatch marks along the network branches indicate the number of mutations. Each color represents a different host. Human (red), Sheep (green), Deer (yellow), Goat (blue), and Cattle (purple).

The networking analysis based on the *COX*1 locus showed a topology similar to the phylogenetic tree. In general, three clusters (*T. vitrinus*, *T. axei*, and *T. colubriformis*) represented long arms due to the number of polymorphisms among the species. The results of network analysis revealed that the sequences of the present study and the sequences from GenBank in relation to *Trichostrongylus* spp. (Figure 4), were organized into three main groups including: *T. vitrinus*, *T. axei*. and *T. colubriformis*. These groups containing sequences of *Trichostrongylus* spp. from animals and humans from over the world. In the present study 26 novel haplotypes of *Trichostrongylus* spp. were identified (25 for *T. colubriformis* and one for *T. vitrinus*). Most of our haplotypes were found as separate haplotypes with low similarity and high diversity to each other, without forming a central haplotype. Furthermore, *Trichostrongylus* spp. from same origin (Fuman's villages), but from different hosts (Sheep, goat, and human) were closely related.

## 4 Discussion

From the public health point of view, zoonotic diseases are an important part of infection control strategies. Zoonotic diseases not only increase public health concerns, but also can disturb animal related industries [34]. Parasitic helminths are common in animal husbandries and negatively affect the growth rate, fertility, and animal products, while increase morbidity and mortality, and induce huge economy loss [35, 36, 37]. Apart from the importance of *Trichostrongylus* spp. in animal husbandry, zoonotic infection due to this helminth has been reported from the world, particularly in regions with close contact between humans and domesticated animals [3].

Human tricostrongylosis is still among the most important STHs in Iran with a prevalence rate ranging from 2% to 18% in north and south‐west provinces [18, 38, 39]. In the current study, *Trichostrongylus* spp., isolated from infected villagers were characterized using *COX1* gene in Fuman district. Fuman is a city in the north of Iran, which is known as an endemic region for trichostrongilosis. The prevalence of *Trichostrongylus* spp. in this region was investigated in both humans and animals during recent years. Sharifdini et al. [18], investigated the prevalence of *Trichostrongylus* spp., in villagers of Fuman city and the prevalence of *Trichostrongylus* spp. in humans was reported as high as 3.05%. The characterized species based on the morphological features and phylogenetical analyses of the ITS gene were *T. colubriformis*, *T. vitrinus, T. longispicularis*, and *T. axei* [18]. In the same region, trichostrongyloid genera were recognized in 83 of 144 fecal samples collected from dairy cattle, sheep, and goats, in which three *Trichostrongylus* species including *T. colubriformis*, *T. vitrinus* and *T. axei* were identified based on morphological characteristics and genetic analyses of the ITS2 gene [9]. In the current study two *T. colubriformis* and *T. vitrinus* were characterized among study population with majority of *T. colubriformis*. *Trichostrongylus colubriformis* seems to be the most prevalent species in humans in the world [5, 16, 40, 41]. This species is the frequently reported species in Iran [17, 18, 42], as well. In addition, *T. colubriformis* is the major species in domesticated animals and is categorized among the highly zoonotic trichostrongylidae [1, 18, 31]. *Trichostrongylus vitrinus* is mainly a parasite that affects ruminants such as sheep, goats, and cattle. Although it is not a common human *Trichostrongylus* spp., it is still capable to being transmitted from animals to humans under certain situation [43, 44]. *Trichostrongylus vitrinus* was previously characterized in human samples of the studied region and seems to be circulated between humans and animals, although it is not a common human *Trichostrongylus* species [18]. Interestingly, both *T. colubriformis* and *T. vitrinus* were reported from a family who were infected by *Trichostrongylus* spp., presenting gastrointestinal symptoms, from Langroud district, east of Guilan province [17]. Cases of trichostrongylosis caused by *T. colubriformis* was also reported in a French family who consumed strawberries from a local garden fertilized by dried manure from local sheep [6]. Taken together, high humidity and suitable environmental conditions in Guilan province together with grazing animals in pasture increase the chance of the eggs to stay alive and transmit to humans. The previous epidemiological data released from Guilan province besides our findings suggests a zoonotic transmission, particularly from sheep and goat together with anthroponotic transmission of *Trichostrongylus* spp. This hypothesis is supported by study performed in Ghana on farmers and their livestock, of which *Trichostrongylus* spp., were isolated from farmers and their animals (goat, cattle, and sheep) [41].

Until now, many investigations have done on the ITS2 fragment to identify the phylogenetic connections of *Trichostrongylus* species [1, 4, 5, 9, 42]. Nevertheless, there are not significant data about discrimination of *Trichostrongylus* spp. based on Mt genome. Sharifdini et al., [18] investigated phylogenetic relationship of *Trichostrongylus* species using *COX*1 gene in Guilan province, northern Iran. In this line, *T. axei* was reported from the cattle, sheep, and goats, while *T. colubriformis* and *T. vitrinus* were only detected in sheep and goats. The genetic divergence within the species of *T. axei*, *T. colubriformis*, and *T. vitrinus* obtained in this study were indicated that *T. axei* and *T. vitrinus* isolated from the sheep and goats were quite similar. The main reason for targeting *COX1* gene in this study was to describe similarity between our isolates with those, which were characterized from animals in same region to explore probable zoonotic transmission. Actually, it was demonstrated that the ITS2 fragment is not discriminative enough to illustrate inter‐ intra‐species similarity/diversity of *Trichostrongylus* spp. [17, 18, 31]. Due to the rare data on the *COX1* gene, particularly those from same region in Iran, in the databases, our molecular analysis was not able to highlight sheep and goat as definitive source of zoonotic transmission, although our findings suggest such transmission cycle.

Genetic diversity analysis is a tool which is employed to assess geographical distribution and host adaptation for infectious agents [45]. In the present analyses, we tested neutrality, which negative values of Tajima's D (Tajima's D: –1.54772; not significant, *p* > 0.10), indicated a rare variation and consistent with population growth or positive selection, while the growth process is normal and the natural selection has not occurred in the population [46]. The current results suggest increased occurring of rare mutations compared to the expectation suggesting new selection due to adaptation to new hosts [47]. Owing to this, although not significant, the negative Tajima's D suggests expansion of *Trichostrongylus* spp., infection from sheep to humans.

Zoonotic pathogens are of public health concern because of their potential food safety implications that can cause emerging diseases. Monitoring zoonotic pathogens in animal populations in a region can provide information to prevent human outbreaks [48]. In addition, the presence of STHs in a community reflects close‐relationship between domesticated animals and humans. In a rural community, manure is usually employed to fertilized farmlands. Nowadays, shifting to chemical fertilizer has decreased the chance of transmission of parasites, particularly STHs to humans. However, the presence of STHs like *Trichostrongylus* spp. in high prevalence rate in a community may indicate employing traditional fertilizers such as night soil. Surveillance systems help detect and respond to new threats before they spread. Due to the public health importance of trichostrongylosis, particularly in endemic regions, having information about the prevalence of *Trichostrongylus* in animals and humans in a region can provide valuable information about the transmission cycle of different species of this parasite between humans and animals [1, 4, 17].

The most important limitation of this study was the short length of mt genome, which was investigated. In addition, we only analyzed *COX1* gene. The pressure of environmental changes and life traits on the host adaptability of nematodes like *Trichostrongylus* spp., may reflects its effects on the mt genome. However, to provide more reliable results and to shape transmission cycle and new hosts, analysis of other mitochondrial genes is needed [49].

## 5 Conclusion

In the current study, *Trichostrongylus* spp., which were collected from villagers in Fuman district, were characterized using targeting and analyzing of a discriminative region of *COX1*. Accordingly, two *T. colubriformis* and *T. vitrinus* were characterized among samples, and molecular analyses suggested a close relationship between our sequences with those from sheep and goats in the same region. These findings strongly suggest the probability of zoonotic transmission of *Trichostrongylus* spp. in Fuman district from domesticated animals. However, due to the rare data about the *COX1* gene, in Iran and the world, this study calls for further molecular investigations in both human and animal sources in endemic regions using discriminative genes such as *COX1*.

## Author Contributions

**Sara Nemati:** investigation, visualization, software, writing – original draft, writing – review and editing. **Hanieh Mohammad Rahimi:** investigation, formal analysis, data curation. **Meysam Sharifdini:** conceptualization, resources. **Hamed Mirjalali:** conceptualization, writing – original draft, writing – review and editing, funding acquisition, formal analysis, supervision, methodology, validation.

## Consent

The authors have nothing to report.

## Conflicts of Interest

The authors declare no conflicts of interest.

## Transparency Statement

The lead author Meysam Sharifdini, Hamed Mirjalali affirms that this manuscript is an honest, accurate, and transparent account of the study being reported; that no important aspects of the study have been omitted; and that any discrepancies from the study as planned (and, if relevant, registered) have been explained.

## Data Availability

The authors confirm that the data supporting the findings of this study are available within the article (and/or) its supplementary materials.
